# Effectiveness of Acupuncture for Early Recovery of Bowel Function in Cancer: A Systematic Review and Meta-Analysis

**DOI:** 10.1155/2017/2504021

**Published:** 2017-12-20

**Authors:** Yi-Hua Liu, Guang-Tong Dong, Yang Ye, Jia-Bin Zheng, Ying Zhang, Hong-Sheng Lin, Xue-Qian Wang

**Affiliations:** ^1^Department of Oncology, Guang'anmen Hospital, China Academy of Chinese Medical Sciences, No. 5 Beixiange Street, Xicheng District, Beijing 100053, China; ^2^Beijing University of Chinese Medicine, No. 11 North Third Ring Road, Chaoyang District, Beijing 100029, China

## Abstract

**Objectives:**

The aim of this study was to evaluate the effects of acupuncture therapy to reduce the duration of postoperative ileus (POI) and to enhance bowel function in cancer patients.

**Methods:**

A systematic search of electronic databases for studies published from inception until January 2017 was carried out from six databases. Randomized controlled trials (RCTs) involving the use of acupuncture and acupressure for POI and bowel function in cancer patients were identified. Outcomes were extracted from each study and pooled to determine the risk ratio and standardized mean difference.

**Results:**

10 RCTs involving 776 cancer patients were included. Compared with control groups (no acupuncture, sham acupuncture, and other active therapies), acupuncture was associated with shorter time to first flatus and time to first defecation. A subgroup analysis revealed that manual acupuncture was more effective on the time to first flatus and the time to first defecation; electroacupuncture was better in reducing the length of hospital stay. Compared with control groups (sham or no acupressure), acupressure was associated with shorter time to first flatus. However, GRADE approach indicated a low quality of evidence.

**Conclusions:**

Acupuncture and acupressure showed large effect size with significantly poor or inferior quality of included trials for enhancing bowel function in cancer patients after surgery. Further well-powered evidence is needed.

## 1. Introduction

Bowel dysfunction has been found to be closely related to worse postoperative quality of life, which is regarded as a major outcome measure in surgical oncology [1, 2]. A temporary impairment of bowel motility lasting within 3–5 days, known as postoperative ileus (POI), is expected after any major surgical procedure, including cancer surgery [3–5]. The clinical manifestations of POI include abdominal pain, nausea, vomiting, and delay in the passage of flatus and stool [6]. A commonly used clinical end point of POI was the time to recovery of bowel function; the primary therapeutic goal in the treatment of POI is to decrease the time to first flatus and defecation [7]. POI is associated with discomfort experienced by patients, delayed patient recovery, prolonged length of hospital stay, and increased healthcare costs [8]. Epidemiological evidence supports the fact that POI is the second most common reason for hospital readmission following surgery; the incidence of POI is 10 to 30% [9, 10]. The annual economic impact of POI management has been estimated to be over 1.5 billion dollars [11].

Since POI is one of the major causes for the delayed recovery of bowel function following cancer resection [12], pharmacological and nonpharmacological therapies have been directed toward alleviating POI [13]. Pharmacological agents such as cyclooxygenase 2 (COX-2) inhibitors, ghrelin agonists, and opioid agonists always bring side effects, such as cardiovascular adverse events and immunosuppressive effects [14]. The laparoscopic technique has been proven to reduce the incidence of POI, while the costs restrict its application. Indeed, no drugs or interventions to prevent POI have been approved by the US Food and Drug Administration [15]. Recently, the Enhanced Recovery after Surgery (ERAS) programs have proposed that measures should aim more at the prevention of POI than its treatment [16, 17], of which acupuncture has become a promising option.

As a nonpharmacological intervention, acupuncture is commonly used for various functional gastrointestinal disorders. A preclinical animal study demonstrated that acupuncture could promote the recovery time of POI by activating the vagus nerve to improve the gastrointestinal tract transit function [18]. Moreover, acupuncture has been widely practiced as an appropriate adjunctive treatment for cancer symptoms [19, 20]. Many researchers have investigated the efficacy of acupuncture therapy for postoperative recovery in cancer patients with POI. However, whether acupuncture has a definite therapeutic effect on POI in cancer patients remains controversial. Two previous systematic reviews broadly for cancer care have examined this issue but did not use meta-analysis [19, 21]. To the best of our knowledge, a meta-analysis has never been performed to address this issue. Hence, we conducted this systematic review and meta-analysis to comprehensively assess the effectiveness of acupuncture and acupressure in preventing POI and enhancing bowel function in cancer patients. 

## 2. Materials and Methods

### 2.1. Protocol and Registration

This meta-analysis follows Preferred Reporting Items for Systematic Reviews and Meta-Analyses Statement (PRISMA) guidelines [22]. The registered study protocol is available on PROSPERO database (identification number: CRD 42016049633).

### 2.2. Data Sources and Search Strategy

The following databases were searched from inception until January 2017: PubMed, EMBASE, Cochrane Central, VIP database for Chinese Technical Periodicals, China National Knowledge Infrastructure database, and Wanfang database. The complete manuscripts of all relevant studies published in English and Chinese were retrieved. The key search terms included [cancer OR tumor OR neoplasm OR oncology] AND [acupuncture OR electroacupuncture OR acupressure] AND [ileus OR intestinal obstruction OR gastrointestinal dysfunction]. The search strategy was modified to suit each database. Additionally, we searched the following databases of ongoing trials: the WHO International Clinical Trials Registry Platform and clinicaltrials.gov. The reference lists of relevant reviews were cross-examined to avoid the risk of missing eligible RCTs.

### 2.3. Inclusion Criteria

The inclusion criteria were as follows: (1) participants: participants underwent surgery were adults aged 18 years or older who were formally diagnosed with cancer. There were no limitations on race, gender, or tumor type. (2) Interventions: manual acupuncture (MA), electroacupuncture (EA), and acupressure were defined as the only forms. Studies that evaluated auricular acupuncture, moxibustion, microacupuncture, and acupuncture point injection were excluded (the methodology and principles in mechanism differ from acupuncture therapy). (3) Controls: no acupuncture, sham acupuncture, and other active control therapies were considered, as no precise definition of placebo acupuncture exists. RCTs comparing acupuncture directly with different types of herbal medications were excluded from this study. (4) Outcomes: the primary outcomes used in this systematic review were time to first flatus and time to defecation, which have been shown to be clinical hallmarks of POI [7]. Secondary outcomes included time to first bowel sounds, opioids consumption, visual analog scale (VAS) pain score, the risk of POI, and length of hospital stay [7, 33]. Studies that did not report at least two aggregate outcomes were excluded. Any adverse events (AE) were measured (if available). (5) Studies: eligible studies had to meet the criteria as follows: only prospective RCTs that evaluated acupuncture to treat POI or bowel dysfunction in cancer patients following surgery were considered; published in a peer-reviewed journal; had original data being independent of other studies. Any conflicts on the eligibility of studies were resolved by consulting a senior researcher (HSL).

### 2.4. Data Extraction

Two reviewers (Yi-Hua Liu and Yang Ye) screened all the records independently. Excluded studies and exclusive reasons were listed. Relevant articles were sorted and cross-examined. The relevant information was extracted using a standardized data extraction form which included first author and publication year, country, study design, baseline characteristics of patients, tumor type, experimental and control type, treatment course, and outcome assessment. The details of intervention and control group were extracted according to the Standards for Reporting Interventions in Controlled Trials of Acupuncture (STRICTA) checklist items. A checklist included theory of acupuncture, needle depth, needle location, name and number of acupuncture points selected, “Deqi” sensation, and duration of treatment sessions [34]. Any discrepancies were discussed and resolved by agreement or by consulting a senior researcher (HSL).

### 2.5. Quality Assessment

The methodological quality was assessed according to the Cochrane Handbook version 5.1.0 [34, 35]. The risk of bias quality was assessed based on seven items: random sequence generation, allocation concealment, blinding of participants and personnel, blinding of outcome assessment, incomplete outcome data, selective reporting, and other bias. After assessing all the domains, the risk of bias was categorized as three levels: low risk, unclear risk, and high risk. Moreover, the Grading of Recommendations Assessment, Development and Evaluation (GRADE) approach was used to facilitate the overall quality of each outcome [36]. The evidence of GRADE was generated using GRADEpro version 3.6 software. Any discrepancies were discussed and resolved by agreement or by consulting a senior researcher (HSL).

### 2.6. Statistical Analysis

Effect sizes were presented as risk ratio (RR) and standardized mean difference (SMD). Dichotomous data were presented as risk ratio (RR) with 95% confidence interval (CI); the standardized mean difference (SMD) with 95% CI was used for effect estimates because outcome measurements are on the various scales. The clinical significance for the SMD was rated (<0.40, 0.40~0.70, >0.70 was, respectively, regarded as low, moderate, and large) according to Cohen's interpretation of effect size. Cochrane *Q* test and *I*^2^ (25%, 50%, and 75% were, respectively, regarded as low, moderate, and high heterogeneity) were used to assess the heterogeneity; *I*^2^ < 50% indicates acceptable heterogeneity [37]. The random effect models were used to give an estimate of the effect because heterogeneity between multistudies and wider intervals has to be considered. Subgroup analysis was designed to investigate the effect of different acupuncture approaches including MA and EA. Sensitivity analysis was carried out for primary outcomes by removing one study a time to investigate the robustness of the review findings. Funnel plots were generated to examine the publication bias and assessed by using Begg's and Egger's tests (if at least 10 trials were available for a meta-analysis) [38]. Cumulative meta-analyses were conducted to analyze how the pooled effect has changed over time. Data analysis was applied using RevMan 5.3 (Cochrane Collaboration) and Stata 12.0 (StataCorp). All *P* values were two sided.

## 3. Results

### 3.1. Search Results

**Figure 1:**
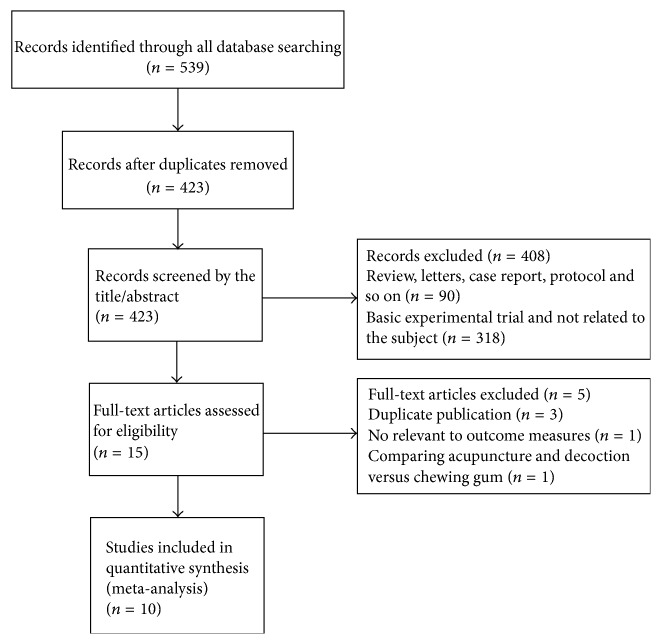
Flow diagram of studies included in the review.

A total of 539 articles were identified in the initial research. Among them, 116 articles were excluded because they were duplicate trials. After screening based on titles and abstracts, 408 were excluded because they were nonclinical trials, case reports, reviews, protocols, or not related to the subject. Of the remaining 15 articles, by reading the full text, 10 articles met our inclusion criteria [23–32]. Flow diagram of the screening process is summarized in Figure 1.

### 3.2. Characteristics of Included Studies

The basic characteristics of the included trials are listed in Table 1. All of the trials were RCTs published from 2008 to 2015. One trial was conducted in the United State [23] and nine conducted in China [24–32].

#### 3.2.1. Participants

A total of 776 cancer patients with sample sizes ranging from 39 to 165 were included in 10 RCTs. Among them, 358 patients received acupuncture treatments, and 418 patients were treated with control. The median age of patients ranged from 53.1 to 68.5. Patients with structural abnormalities were excluded in each trial. Underlying malignancies included gastric cancer [24, 32], colorectal cancer [25–31], and miscellaneous cancer [23].

#### 3.2.2. Interventions

MA was used in five trials [23, 24, 27, 29, 31], EA was used in three trials [25, 28, 30], and acupressure was used in two trials [26, 32]. All trials mentioned the starting time of the intervention. The most commonly used acupuncture points were ST-36 (ten trials) and SP-6 (five trials). Total treatment sessions ranged from 3 days to 10 days, and intervention dose ranged from 3 min to 45 min.

#### 3.2.3. Outcome Measures

Most trials adopted the time to first flatus and time to first defecation as the primary outcome (9/10). For the secondary outcomes, four trials evaluated VAS pain score [23, 25, 28, 29] and opioids consumption [23, 28–30]. Three trials evaluated the risk of POI [23, 25, 28], the length of hospital stay [23, 28, 30], and time to first bowel sound [27, 30, 31]. Seven trials concluded the efficacy of acupuncture and acupressure on the improvement of primary outcome [24, 26–28, 30–32], while three trials suggested no effects [23, 25, 29]. For studies with two control groups, we divided the shared intervention evenly between groups as described in the Cochrane Handbook [37]. The details of acupuncture therapy assessed by STRICTA were presented in Supplementary File 3.

### 3.3. Risk of Bias and Level of Evidence

**Figure 2:**
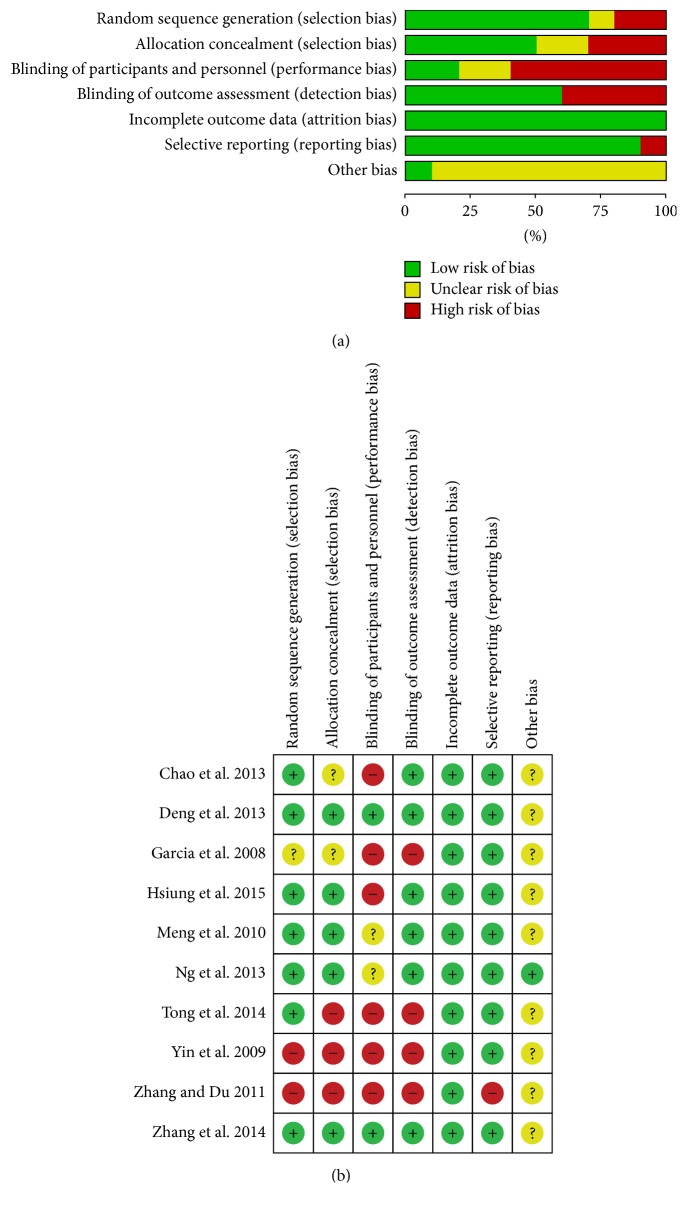
(a) Risk of bias graph: review author's judgements about each risk of bias item presented as percentages across all included studies. (b) Risk of bias summary: review author's judgements about each risk of bias item for each included study.

Although all ten trials reported that they utilized randomized control procedures, only seven trials described the generation of random sequences [25, 26, 28–32] while five trials described the method of allocation concealment [25, 28–30, 32]. In the trials comparing acupuncture with sham or no acupuncture that did not insert needles into skin, patients blinding was not possible and likely to be a risk in most RCTs. In most of the studies, the use of intention-to-treat analysis was unclear. One trial [25] reported a number of dropouts (10/85); due to the dropouts evenly distributed across intervention and control group, the rate below 15% would be unlikely to affect the estimates. The total quality of the data was low according to Cochrane risk of bias tools. A summary of risks of bias is presented in Figure 2. The GRADE approach was used to quantify the effects and level of evidence. According to the GRADE assessment, the quality of evidence for most outcomes was from very low to low. The imprecision domain of outcomes was downgraded due to methodological limitations and small sample sizes. The summary of evidence generated using GRADEPRO is presented in Table 2.

### 3.4. Meta-Analysis Results

We combined the data using a random effect model in the following comparison: (1) acupuncture (MA and EA) versus control; (2) acupressure versus control.

#### 3.4.1. Effects of Acupuncture Treatment

**Figure 3:**
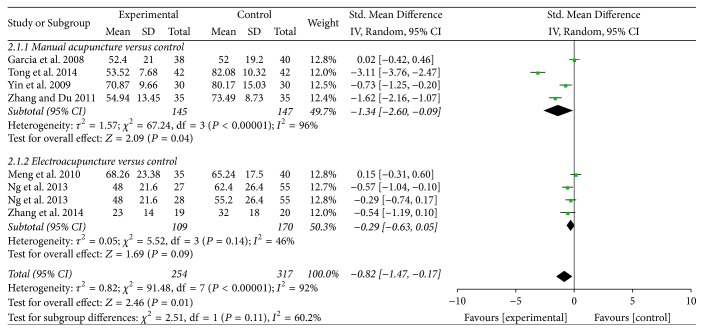
Forest plot of acupuncture treatment versus control group: time to first flatus.

*Time to First Flatus.* Seven trials contributed to the combined calculation of this outcome [23–25, 27, 28, 30, 31]. Pooled results indicated that acupuncture treatment was associated with a significant benefit in time to first flatus (SMD = −0.82, 95% CI: −1.47 to −0.17, *P* = 0.01; *I*^2^ = 92%) (Figure 3). A subgroup analysis was conducted to explore whether heterogeneity could be partially explained by different types of acupuncture treatment. Our results indicated that MA was associated with shorter time to first flatus (SMD = −1.34, 95% CI: −2.60 to −0.09, *P* = 0.04), while EA found no significant difference (SMD = −0.29, 95% CI: −0.63 to −0.05, *P* = 0.09). Subgroup analyses were also conducted according to the control group, cancer type (e.g., gastric cancer and colorectal cancer), and different type of randomization; no difference in overall conclusion was observed, while heterogeneity was not resolved (Supplementary File 1). The subgroup analysis based on the type of acupuncture points used revealed that ST-36 (Zusanli) plus SP-6 (Sanyinjiao) treatment significantly reduced the time to first flatus (SMD = −0.62, 95% CI: −1.14 to −0.10, *P* = 0.02), while ST-36 treatment found no significant difference (SMD = −1.16, 95% CI: −3.08 to −0.75, *P* = 0.23) (Supplementary File 1E).

**Figure 4:**
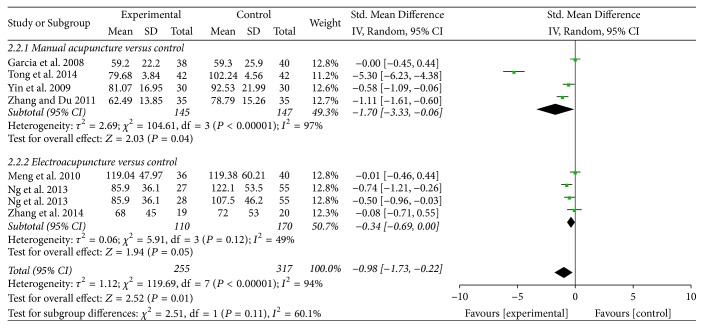
Forest plot of acupuncture treatment versus control group: time to first defecation.

*Time to First Defecation*. Seven trials contributed to the combined calculation of this outcome [23–25, 27, 28, 30, 31]. Pooled results indicated that acupuncture treatment was associated with shorter time to first defecation (SMD = −0.98, 95% CI: −1.73 to −0.22, *P* = 0.01; *I*^2^ = 94%) (Figure 4). A subgroup analysis was conducted according to different types of acupuncture treatment. Our results indicated that MA was associated with shorter time to first defecation (SMD = −1.70, 95% CI: −3.33 to −0.06, *P* = 0.04), while EA found no significant difference (SMD = −0.34, 95% CI: −0.69 to 0.00, *P* = 0.05). Subgroup analysis was also conducted according to the control group, cancer type, and type of randomization, while heterogeneity was not resolved (Supplementary File 1). The subgroup analysis based on the type of acupuncture points used revealed that ST-36 plus SP-6 treatment significantly reduced the time to first defecation (SMD = −0.57, 95% CI: −0.93 to −0.22, *P* = 0.03), while ST-36 treatment found no significant difference (SMD = −1.77, 95% CI: −4.47 to −0.94, *P* = 0.20) (Supplementary File 1F).

**Figure 5:**
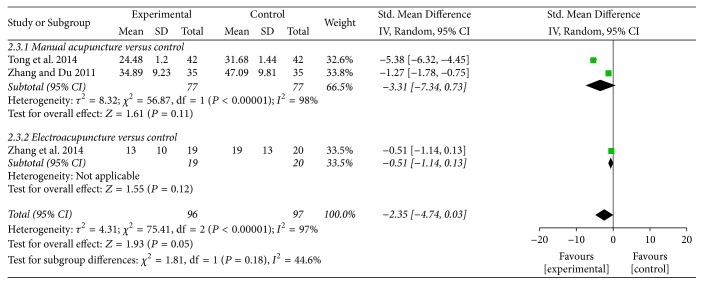
Forest plot of acupuncture treatment versus control group: time to first bowel sound.

*Time to First Bowel Sounds. *Three trials contributed to the combined calculation of this outcome [27, 30, 31]. Pooled results indicated that acupuncture treatment was associated with a shorter time to first bowel sounds, but the difference was not statistically significant (SMD = −2.35, 95% CI: −4.74 to 0.03, *P* = 0.05; *I*^2^ = 97%) (Table 2, Figure 5). The potential source of heterogeneity could not be explained by subgroup analysis.

**Figure 6:**
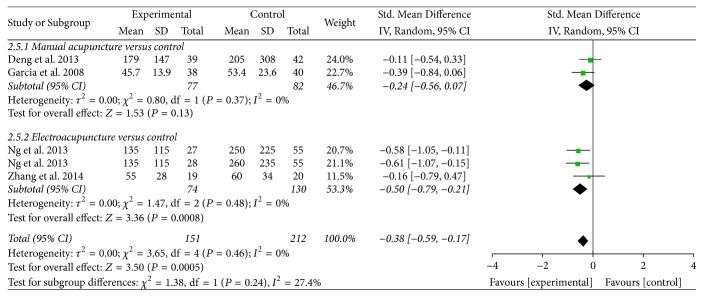
Forest plot of acupuncture treatment versus control group: opioids consumption.

**Figure 7:**
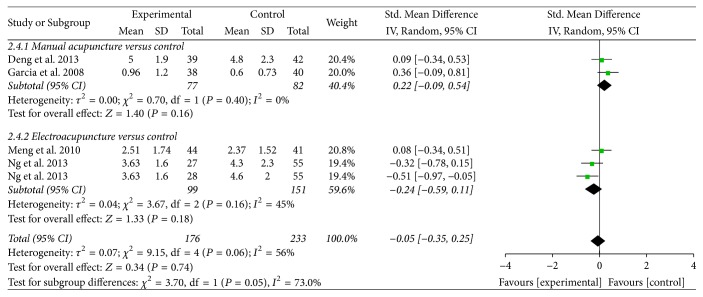
Forest plot of acupuncture treatment versus control group: pain score.

*Opioids Consumption and Pain Score. *Four trials reported on these outcomes of opioids consumption [23, 28–30] and pain score [23, 25, 28, 29]. Pooled results indicated that acupuncture treatment was associated with fewer opioids consumption (SMD = −0.38, 95% CI: −0.59 to −0.17, *P* = 0.0005; *I*^2^ = 0%) (Table 2, Figure 6). The subgroup analysis showed that EA was associated with fewer opioids consumption (SMD = −0.50, 95% CI: −0.79 to −0.21, *P* = 0.0008), while MA found no significant difference (SMD = −0.24, 95% CI: −0.56 to 0.07, *P* = 0.13). The subgroup analysis based on the type of acupuncture points used revealed that ST-36 plus SP-6 treatment significantly reduced the opioids consumption (SMD = −0.41, 95% CI: −0.64 to −0.18, *P* = 0.0005), while ST-36 treatment found no significant difference (SMD = −0.16, 95% CI: −0.79 to −0.47, *P* = 0.62) (Supplementary File 1G). Compared with control group, acupuncture treatment was not superior on pain score (SMD = −0.05, 95% CI: −0.35 to 0.25, *P* = 0.74; *I*^2^ = 56%) (Table 2, Figure 7).

**Figure 8:**
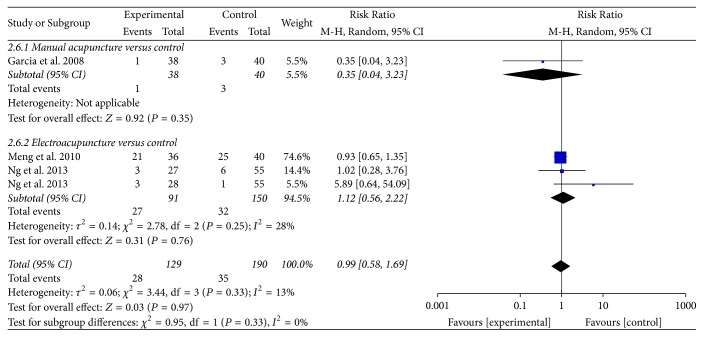
Forest plot of acupuncture treatment versus control group: risk of POI.

**Figure 9:**
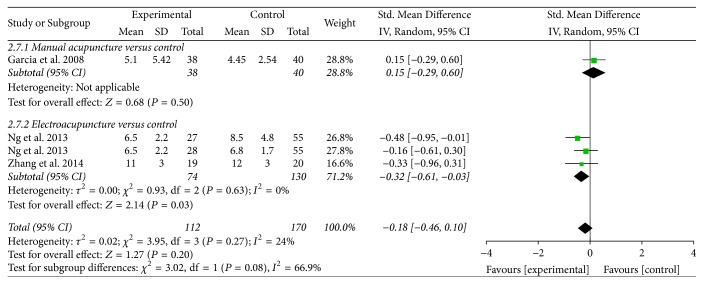
Forest plot of acupuncture treatment versus control group: length of hospital stay.

*Postoperative Ileus and Length of Hospital Stay. *Three trials reported on outcomes of POI [23, 25, 28] and length of hospital stay [23, 28, 30]. Compared with control group, acupuncture treatment was not superior in reducing the risk of POI (RR = 0.99, 95% CI: 0.58 to 1.69, *P* = 0.97; *I*^2^ = 13%) (Table 2, Figure 8) and was not superior in reducing the length of hospital stay (SMD = −0.18, 95% CI: −0.46 to 0.10, *P* = 0.20; *I*^2^ = 24%) (Table 2, Figure 9). The subgroup analysis showed that EA was associated with shorter length of hospital stay (SMD = −0.32, 95% CI: −0.61 to 0.03, *P* = 0.03).

#### 3.4.2. Effects of Acupressure Treatment

**Figure 10:**
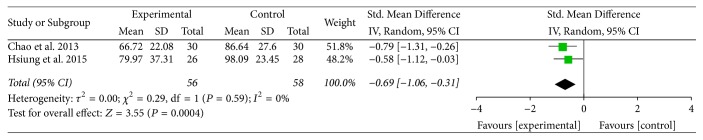
Forest plot of acupressure treatment versus control group: time to first flatus.

**Figure 11:**
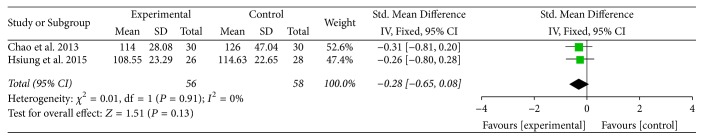
Forest plot of acupressure treatment versus control group: time to first defecation.

*Time to First Flatus and Time to First Defecation. *Two trials contributed to the combined calculation of these outcomes [26, 32]. Pooled results indicated that acupressure was associated with a significant benefit in time to first flatus (SMD = −0.69, 95% CI: −1.06 to −0.31, *P* = 0.0004; *I*^2^ = 0%) (Table 2, Figure 10). Acupressure was associated with no significant benefits in time to first defecation (SMD = −0.28, 95% CI: −0.65 to 0.08, *P* = 0.13) (Table 2, Figure 11).

### 3.5. Adverse Events

Of the ten included RCTs, only four trials assessed adverse effects and the others did not. One trial [23] mentioned that no serious adverse events related to acupuncture therapy occurred. Three trials [25, 28, 32] reported that there were no adverse events related to acupuncture therapy reported.

### 3.6. Sensitivity Analysis

A sensitivity analysis was undertaken by removing one study at a time to find the explanation and investigating the influence of each study on the overall risk estimate. The result of time to first flatus and time to first defecation was not significantly affected by omission of a single study (Supplementary File 2). The combined SMD per change in time to first flatus ranged from −0.49 (95% CI: −0.92 to −0.07) to −0.96 (95% CI: −1.66 to −0.21); time to first defecation ranged from −0.42 (95% CI: −0.74 to −0.11) to −1.12 (95% CI: −1.99 to −0.25). After carefully reviewing included trials, we can reasonably conclude that these heterogeneities cannot impair the overall effect size of acupuncture treatment.

### 3.7. Publication Bias

Funnel plots were not applied to investigate the publication bias since the number of included studies was limited (less than 10 trials).

### 3.8. Cumulative Meta-Analysis

Cumulative meta-analysis indicated that a statistically significant effect of acupuncture treatment on the time to first flatus was first observed after the publication of the 7th trial in 2014, while the time to first defecation was first observed after the 5th trial in 2013. The subsequent trials did not increase the precision of the pooled results, and no change occurred in the trend of the intervention effect (Supplementary File 2).

## 4. Discussion

### 4.1. Summary of Evidence

The current meta-analysis of 10 trials demonstrated that acupuncture was associated with reduced time to first flatus, time to first defecation, and fewer opioids consumption in cancer patients after surgery. Moreover, acupressure was more effective than other comparators for the time to first flatus. These findings indicated that acupuncture and acupressure tend to have better benefits for the recovery of bowel function than sham acupuncture/acupressure, no acupuncture/acupressure, or other active control therapy (such as acupuncture plus Chinese herbal medicine versus Chinese herbal medicine). However, the results might differ by the type of acupuncture and acupuncture point. Subgroup analysis suggested that MA was more effective in the improvement of time to first flatus and time to first defecation, while EA was associated with reduced the length of hospital stay. ST-36 plus SP-6 treatment was associated with reduced time to first flatus, time to first defecation, and opioids consumption, while ST-36 treatment found no significant difference. However, these subgroups contain other acupuncture points except ST-36 and SP-6. Due to the confounding factors of other acupuncture points, the comprehensive conclusion of whether the result varied by acupuncture points is difficult to draw. In the sensitive analysis of primary outcomes, omitting any trial cannot impair the overall effect size of acupuncture.

Time to first flatus and time to first defecation are essential components in the assessment of gastrointestinal dysfunction and POI [7]. These outcomes are commonly accepted as the clinical endpoint of POI and valuable indexes for evaluating the effect of interventions [39]. The decreased time to first flatus and time to first defecation after interventions indicate the improvement of bowel function. However, currently available evidence for these indicators is very limited. Nine RCTs with a small sample size assessed these measurements, while no trials mention the long-term effect of acupuncture and acupressure. Moreover, substantial heterogeneity was a limitation of the analysis of acupuncture for these measurements. The variety of intervention and control groups may have contributed to heterogeneity. But subgroup analysis (according to the type of intervention, type of control group, and type of cancer) did not totally explain the heterogeneity. It should also be emphasized through that time to first flatus is difficult to assess accurately (self-reported outcomes) [40]. Without clear measures, separating the specific effects of needling from nonspecific therapeutic factors is challenging.

The optimal management of postoperative pain is an important component of postoperative recovery after surgical resection [41]. Anesthetics and opioid analgesics used in the postoperative period are believed to be major factors that contribute to delayed bowel function [41]. The reduction of VAS pain score and opioids consumption can also indicate the effect of interventions for bowel function. The results of this meta-analysis indicated that acupuncture could significantly decrease the use of opioids consumption, which is consistent with previous studies supporting the relationship between acupuncture and postoperative pain [42]. The heterogeneity was acceptable in the analysis of these measurements. However, the included four trials did not report data of dropouts and intention-to-treat analysis.

In addition, POI due to oncological surgery greatly influences patients' length of hospital stay [8]. The overall effect of risk of POI and length of hospital stay showed no significant difference compared with control groups (sham acupuncture and no acupuncture). Subgroup analysis supported a benefit of EA on the length of hospital stay. Two trials of EA exactly reported the process of the randomization and allocation concealment. Meanwhile, the heterogeneity was low in the analysis of these measurements. The finding was remarkable, but the evidence was still limited.

Since the quality of outcomes determined by GRADE approach was low to very low, the currently available evidence is inadequate to make a firm conclusion on the issue of effectiveness by using acupuncture and acupressure.

### 4.2. Potential Mechanism of Acupuncture

POI is a common gastrointestinal problem following surgery. Risk factors for POI include long time of surgery, hemorrhage, and extensive manipulations of abdominal cavity [43]. Since these conditions frequently occur in cancer patients, it can be expected that cancer patients would have more risk of developing POI than others. A variety of strategies have been proposed to reduce POI. Nonpharmacological techniques such as laparoscopic surgery and fast-track recovery programs are primarily aimed at smaller incisions, reduced pain, and improved perioperative care management; pharmacological agents such as cyclooxygenase 2 (COX-2) inhibitors, ghrelin agonists, and opioid agonists focus on decreasing inflammation or acting on *μ*-opioid-receptor [14]. Since no strategies have been completely successful in preventing postoperative ileus, complementary and alternative therapies may be used as an adjunct to symptom management [14, 44, 45].

Acupuncture is a widely used complementary and alternative treatment for alleviating symptoms and improving the quality of life in cancer patients, and it is considered an effective adjuvant approach for various gastrointestinal diseases [19, 20]. A commonly used clinical end point of POI was the time to recovery of gastrointestinal function. Although the exact mechanism of how acupuncture may reduce POI in cancer patients is unknown, acupuncture has been shown to improve gastrointestinal dysrhythmia [46, 47], secretion [48], accelerate solid gastric emptying [49], and restore impaired gastrointestinal motility mediated via the cholinergic pathway [50]. Extensive research indicates that acupuncture has the potential to treat gastrointestinal disorders by regulating the gastrointestinal barrier, visceral sensitivity, and the brain-gut axis [51]. A pilot study found that acupuncture has the potential effect on reducing the duration of POI after gastric cancer surgery, by the earlier recovery of small bowel movement [52]. A recent preclinical study has revealed that EA administered at ST36 promotes the recovery time of POI by exciting nucleus of the solitary tract neurons [18]. Thus, for an intervention that is effective and of low cost and has few side effects, acupuncture is worth clinical generalization and application.

### 4.3. Comparison with Other Studies

A previous systematic review evaluated the use of acupuncture broadly for cancer care but did not provide a determined result for POI in cancer patients. In this study, potential available RCTs were assessed as having a high risk of bias, and no trials met their inclusion criteria [19]. Another systematic review suggested that acupuncture for cancer patients with POI presented no significant difference within the intergroups [21]. Indeed, no meta-analysis has been performed before. The current updated meta-analysis reveals some new findings. First, due to the similar mechanism of action, we included additional intervention type of acupressure to assess its effect on POI in cancer patients. The results of this meta-analysis suggested that acupuncture was associated with shorter time to first flatus and time to defecation compared with all control interventions, which differed from the previous reviews. Second, this review managed to identify more RCTs to explore any available evidence. Two previous RCTs demonstrated no significant difference of acupuncture compared with sham or no acupuncture in cancer patients with POI. Meng et al. [25] acknowledged that the use of epidural anesthesia might have diminished the possible effects of acupuncture mediated by neural mechanisms; Garcia et al. [23] did not mention the randomization and allocation concealment. Then, Ng et al. [28] conducted a larger and more rigorous randomized study, excluded patients who had received anesthesia or analgesia, and minimized the risk of randomization and allocation concealment. They found that EA reduced the duration of POI, and opioids consumption, compared with sham or no acupuncture in colorectal cancer. Most other results of recent RCTs are consistent with this trial. Cumulative meta-analysis indicated that the subsequent trials did not increase the precision of the pooled positive results.

### 4.4. Limitations

This meta-analysis has a number of limitations. One obvious limitation is the diversity of control interventions, acupuncture manipulation (needle location, treatment duration, and frequency); therefore, definite conclusions regarding these confounders could not be drawn. High-quality trials with large sample size are needed to provide stronger evidence. Further, a lack of standardized study design and outcome measures might affect the reliability of findings. Future trials should follow the CONSORT statement or STRICTA recommendations [34]. In addition, the databases considered in this review were limited to English and Chinese literature which might contribute to bias and limit our findings.

## 5. Conclusion

In conclusion, the present findings suggest that acupuncture and acupressure demonstrate better efficacy for cancer patients on the recovery of bowel function compared with control interventions. However, the effect size of acupuncture treatment might be limited by the inferior quality of included trials. Further rigorous studies with adequate power are needed to determine the effectiveness of acupuncture in cancer patients with bowel dysfunction and POI.

## Figures and Tables

**Table 1:** Basic characteristics of the included studies.

Study (years)	Cancer type	Intervention group	Control group	The main points	Treatment course	Main outcome	Results	Sample size (*I/N*)
Garcia et al. 2008 [23]	NR	MA	NA	ST-36 (Zusanli), ST-25 (Tianshu), LI-4 (Hegu), SP-6 (Sanyinjiao), CV-6 (Qihai), CV-12 (Zhongwan)	20 min, 2x/d for 4 d, on po-day 1	(1) TFF/TFD (2) OC/LH (3) Pain	No reduction	78 (38/40)

Yin et al. 2009 [24]	Gastric cancer	MA + decoction	decoction	ST-36 (Zusanli), SP-6 (Sanyinjiao), PC-6 (Neiguan), LR3 (Taichong)	45 min, 1x/d for 7 d, on po-day 3	(1) TFF/TFD (2) TFB	Decreased TFF/TFD and TFB	60 (30/30)

Meng et al. 2010 [25]	Colorectal cancer	EA	NA	ST-36 (Zusanli), ST-37 (Shangjuxu), TE-6 (Zhigou), GB-34 (Yanglingquan)	20 min, 1x/d for 6 d, on po-day 1	(1) TFF/TFD (2) Pain	No reduction	85 (44/41)

Chao et al. 2013 [26]	Colorectal cancer	AC	SAC	ST-36 (Zusanli)	3 min. 3x/d for 5 d, on po-day 1	(1) TFF/TFD	Decreased TFF	60 (30/30)

Zhang and Du 2011 [27]	Colorectal cancer	MA	NA	ST-36 (Zusanli), SP-6 (Sanyinjiao), SP-9 (Yinlingquan), ST-37 (Shangjuxu), ST-39 (Xiajuxu)	45 min, 1x/d for 10 d, on po-day 1	(1) TFF/TFD (2) TFB	Decreased TFF/TFD and TFB	70 (35/35)

Ng et al. 2013 [28]	Colorectal cancer	EA	NA/SEA	ST-36 (Zusanli), SP-6 (Sanyinjiao), LI-4 (Hegu), TE-6 (Zhigou)	20 min, 1x/d for 4 d, on po-day 1	(1) TFF/TFD (2) TFB (3) Pain (4) OC	Decreased TFD/DH	165 (55/55/55)

Deng et al. 2013 [29]	Colorectal cancer	MA	SMA	ST-36 (Zusanli), PC-6 (Neiguan), LI-4 (Hegu), SP-6 (Sanyinjiao), SP-9 (Yinlingquan), ST-25 (Tianshu), auricular shenmen	30 min, 2x/d for 3 d, on po-day 1	(1) OC (2) Pain	No reduction	81 (39/42)

Zhang et al. 2014 [30]	Colorectal cancer	EA	SEA	ST-36 (Zusanli)	30 min, 1x/d for 4 d, on po-day 1	(1) TFF/TFB (2) TFD (3) OC/LH	Decreased TFF and TFB	39 (19/20)

Tong et al. 2014 [31]	Rectal cancer	MA	NA	ST-36 (Zusanli), PC-6 (Neiguan), ST-37 (Shangjuxu), SP-4 (Gongsun)	30 min, 1x/d, on po-day 1	(1) TFF/TFD (2) TFB	Decreased TFF/TFD and TFB	84 (42/42)

Hsiung et al. 2015 [32]	Gastric cancer	AC	NAC	ST-36 (Zusanli), PC-6 (Neiguan)	12 min. 1x/d for 3 d, on po-day 1	(1) TFF/TFD (2) Pain	Decreased TFF	54 (26/28)

MA: manual acupuncture; SMA: sham manual acupuncture; EA: electroacupuncture; SEA: sham electroacupuncture; NA: no acupuncture or usual care; AC: acupressure; SAC: sham acupressure; NAC: no acupressure or usual care; I/C: intervention group versus control group; D: days; Min: minutes; PO: postoperative; NR: not reported; 2x/d for 3d, twice daily, 3 days; LH: length of hospital stay; OC: opioids consumption; TFF: time to first flatus; TFB: time to first bowel sound; TFD: time to first defecation.

**Table 2:** Level of evidence (GRADE).

Outcomes	Illustrative assumed risk	comparative risks^*∗*^ (95% CI)	Relative effect (95% CI)	Number of participants (studies)	Quality of the evidence	Comments
Time to first flatus		The mean time to first flatus in the intervention groups was *0.82 standard deviations lower* (1.47 to 0.17 lower)		571 (8 studies)	⊕⊕ ⊝⊝ *low*^1^	SMD −0.82 (−1.47 to −0.17)

Time to first defecation		The mean time to first defecation in the intervention groups was *0.98 standard deviations lower* (1.73 to 0.22 lower)		572 (8 studies)	⊕⊕ ⊝⊝ *low*^1^	SMD −0.98 (−1.73 to −0.22)

Pain score		The mean pain score in the intervention groups was *0.05 standard deviations lower* (0.35 lower to 0.25 higher)		409 (5 studies)	⊕ ⊝⊝⊝ *very low*^1^	SMD −0.05 (−0.35 to 0.25)

Opioids consumption		The mean opioids consumption in the intervention groups was *0.38 standard deviations lower* (0.59 to 0.17 lower)		363 (5 studies)	⊕ ⊝⊝⊝ *very low*^1^	SMD −0.38 (−0.59 to −0.17)

Length of hospital stay		The mean length of hospital stay in the intervention groups was *0.18 standard deviations lower* (0.46 lower to 0.1 higher)		282 (4 studies)	⊕ ⊝⊝⊝ *very low*^1^	SMD −0.18 (−0.46 to 0.1)

Postoperative ileus	*184 per 1000*	*184 per 1000* (90 to 338)	*RR 1* (0.58 to 1.69)	319 (4 studies)	⊕ ⊝⊝⊝ *very low*^1^	
	*92 per 1000*	*92 per 1000* (43 to 186)				

Time to first bowel sound		The mean time to first bowel sound in the intervention groups was *2.35 standard deviations lower* (4.74 lower to 0.03 higher)		193 (3 studies)	⊕ ⊝⊝⊝ *very low*^1^	SMD −2.35 (−4.74 to 0.03)

Acupressure-time to first flatus		The mean acupressure-time to first flatus in the intervention groups was *0.69 standard deviations lower* (1.06 to 0.31 lower)		114 (2 studies)	⊕ ⊝⊝⊝ *very low*^1^	SMD −0.69 (−1.06 to 0.31)

Acupressure-time to defecation		The mean acupressure-time to defecation in the intervention groups was *7.75 lower* (18.15 lower to 2.65 higher)		114 (2 studies)	⊕ ⊝⊝⊝ *very low*^1^	SMD −0.28 (−0.65 to 0.08)

^*∗*^The basis for the assumed risk (e.g., the median control group risk across studies) is provided in footnotes. The corresponding risk (and its 95% confidence interval) is based on the assumed risk in the comparison group and the relative effect of the intervention (and its 95% CI); CI: confidence interval; RR: risk ratio; high quality: further research is very unlikely to change our confidence in the estimate of effect; moderate quality: further research is likely to have an important impact on our confidence in the estimate of effect and may change the estimate; low quality: further research is very likely to have an important impact on our confidence in the estimate of effect and is likely to change the estimate; very low quality: we are very uncertain about the estimate.

## Supplementary Materials

Supplementary Materials Supplementary file 1. Subgroup analysis. Supplemental file 1A–D: primary outcomes of acupuncture treatment: subgroup analysis by cancer type and various control interventions. Supplemental file 1A: Forest plots of outcome “Time to first flatus” Subgroup: cancer type. Supplemental file 1B: Forest plots of outcome “Time to first defecation” Subgroup: cancer type. Supplemental file 1C: Forest plots of outcome “Time to first flatus” Subgroup: control type. Supplemental file 1D: Forest plots of outcome “Time to first defecation” Subgroup: control type. Supplemental file 1E–G: primary outcomes of acupuncture treatment: subgroup analysis by acupuncture points. Supplemental file 1E: Forest plots of outcome “Time to first flatus” Subgroup: acupuncture points. Supplemental file 1F: Forest plots of outcome “Time to first defecation” Subgroup: acupuncture points. Supplemental file 1G: Forest plots of outcome “Opioids consumption” Subgroup: acupuncture points. Supplementary file 2. Sensitivity analysis and Cumulative meta-analysis. Supplementary file 2. Sensitivity analysis and Cumulative meta-analysis. Supplemental file 2A-B. Sensitivity analyses of the comparison between acupuncture and control group. a: Time to first flatus; b: Time to first defecation. Supplemental file 2A. Sensitivity analysis for time to first flatus between acupuncture and control group. Supplemental file 2B. Sensitivity analysis for time to first defecation between acupuncture and control group. Supplemental file 2C-D. Cumulative meta-analysis of the comparison between acupuncture and control group. c Time to first flatus; d Time to first defecation. Supplemental file 2C: Cumulative meta-analysis of the comparison between acupuncture and control group: time to first flatus. Supplemental file 2D: Cumulative meta-analysis of the comparison between acupuncture and control group: time to first defecation. Supplementary file 3. Reporting quality of RCTs based on STRICTA.
