# Supplementary material for: Effectiveness of Acupuncture for Early Recovery of Bowel Function in Cancer: A Systematic Review and Meta-Analysis
Source: Evid Based Complement Alternat Med. 2017 Dec 20;2017:2504021. doi: 10.1155/2017/2504021 (PMC5750515; doi:10.1155/2017/2504021)
Supplement: Supplementary Materials — Supplementary file 1. Subgroup analysis. Supplemental file 1A–D: primary outcomes of acupuncture treatment: subgroup analysis by cancer type and various control interventions. Supplemental file 1A: Forest plots of outcome “Time to first flatus” Subgroup: cancer type. Supplemental file 1B: Forest plots of outcome “Time to first defecation” Subgroup: cancer type. Supplemental file 1C: Forest plots of outcome “Time to first flatus” Subgroup: control type. Supplemental file 1D: Forest plots of outcome “Time to first defecation” Subgroup: control type. Supplemental file 1E–G: primary outcomes of acupuncture treatment: subgroup analysis by acupuncture points. Supplemental file 1E: Forest plots of outcome “Time to first flatus” Subgroup: acupuncture points. Supplemental file 1F: Forest plots of outcome “Time to first defecation” Subgroup: acupuncture points. Supplemental file 1G: Forest plots of outcome “Opioids consumption” Subgroup: acupuncture points. Supplementary file 2. Sensitivity analysis and Cumulative meta-analysis. Supplementary file 2. Sensitivity analysis and Cumulative meta-analysis. Supplemental file 2A-B. Sensitivity analyses of the comparison between acupuncture and control group. a: Time to first flatus; b: Time to first defecation. Supplemental file 2A. Sensitivity analysis for time to first flatus between acupuncture and control group. Supplemental file 2B. Sensitivity analysis for time to first defecation between acupuncture and control group. Supplemental file 2C-D. Cumulative meta-analysis of the comparison between acupuncture and control group. c Time to first flatus; d Time to first defecation. Supplemental file 2C: Cumulative meta-analysis of the comparison between acupuncture and control group: time to first flatus. Supplemental file 2D: Cumulative meta-analysis of the comparison between acupuncture and control group: time to first defecation. Supplementary file 3. Reporting quality of RCTs based on STRICTA. [file 2504021.f1.docx]

Evidence-based Complementary and Alternative Medicine Supplementary Materials

**Effectiveness of Acupuncture for Early Recovery of Bowel Function in Cancer: A Systematic Review and Meta-Analysis**

Yi-Hua Liu, Yang Ye, Jia-Bin Zheng, Ying Zhang, Hong-Sheng Lin

**Correponding author:**

Hong-Sheng Lin

Department of Oncology, Guang'anmen Hospital, China Academy of Chinese Medical Sciences, Beixiange 5, Xi Cheng District, Beijing, China

Email: dr[linhongsheng@126.com](mailto:linhongsheng@126.com)

**Supplementary file1: Subgroup analysis.**

**Supplemental file 1A-D:** primary outcomes of acupuncture treatment: subgroup analysis by cancer type and various control interventions.

**

**

Supplemental file 1A: Forest plots of outcome “Time to first flatus” Subgroup: cancer type.

**

**

Supplemental file 1B: Forest plots of outcome “Time to first defecation” Subgroup: cancer type.

**

**

Supplemental file 1C: Forest plots of outcome “Time to first flatus” Subgroup: control type.

**

**

Supplemental file 1D: Forest plots of outcome “Time to first defecation” Subgroup: control type.

**Supplemental file 1E-G:** primary outcomes of acupuncture treatment: subgroup analysis by acupuncture points.

**
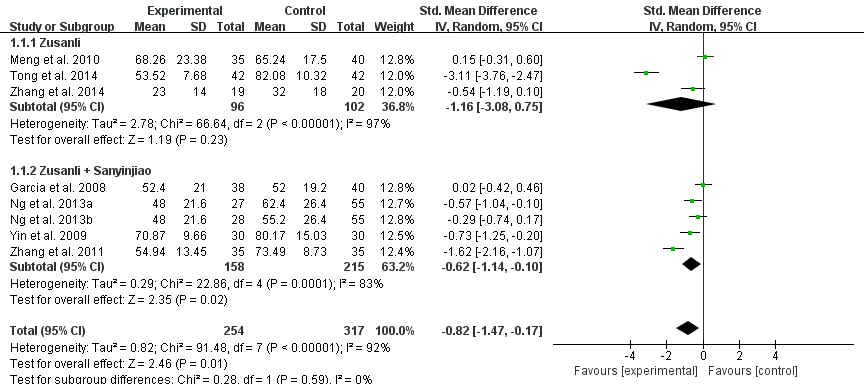
**

Supplemental file 1E: Forest plots of outcome “Time to first flatus” Subgroup: acupuncture points.

**
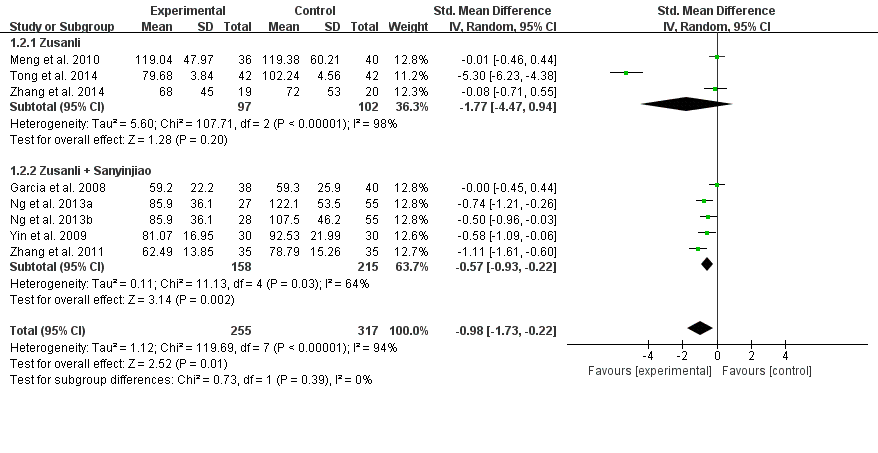
**

Supplemental file 1F: Forest plots of outcome “Time to first defecation” Subgroup: acupuncture points.

**
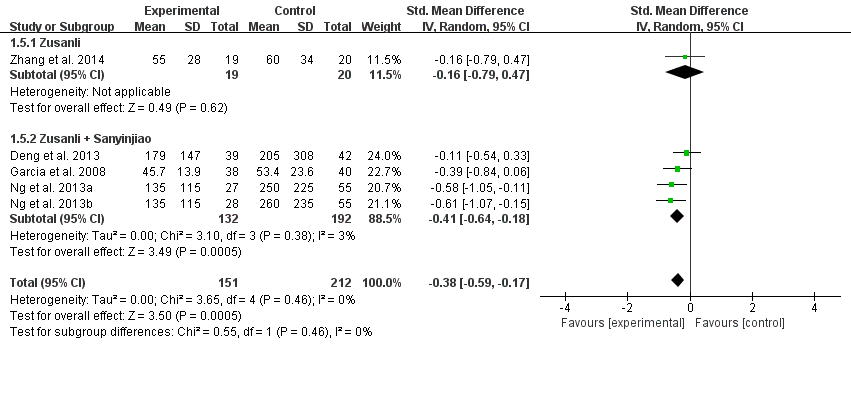
**

Supplemental file 1G: Forest plots of outcome “Opioids consumption” Subgroup: acupuncture points.

**Supplementary file2: Sensitivity analysis and Cumulative meta-analysis.**

**Supplemental file 2A-B.** Sensitivity analyses of the comparison between acupuncture and control group. **a** Time to first flatus; **b** Time to first defecation.


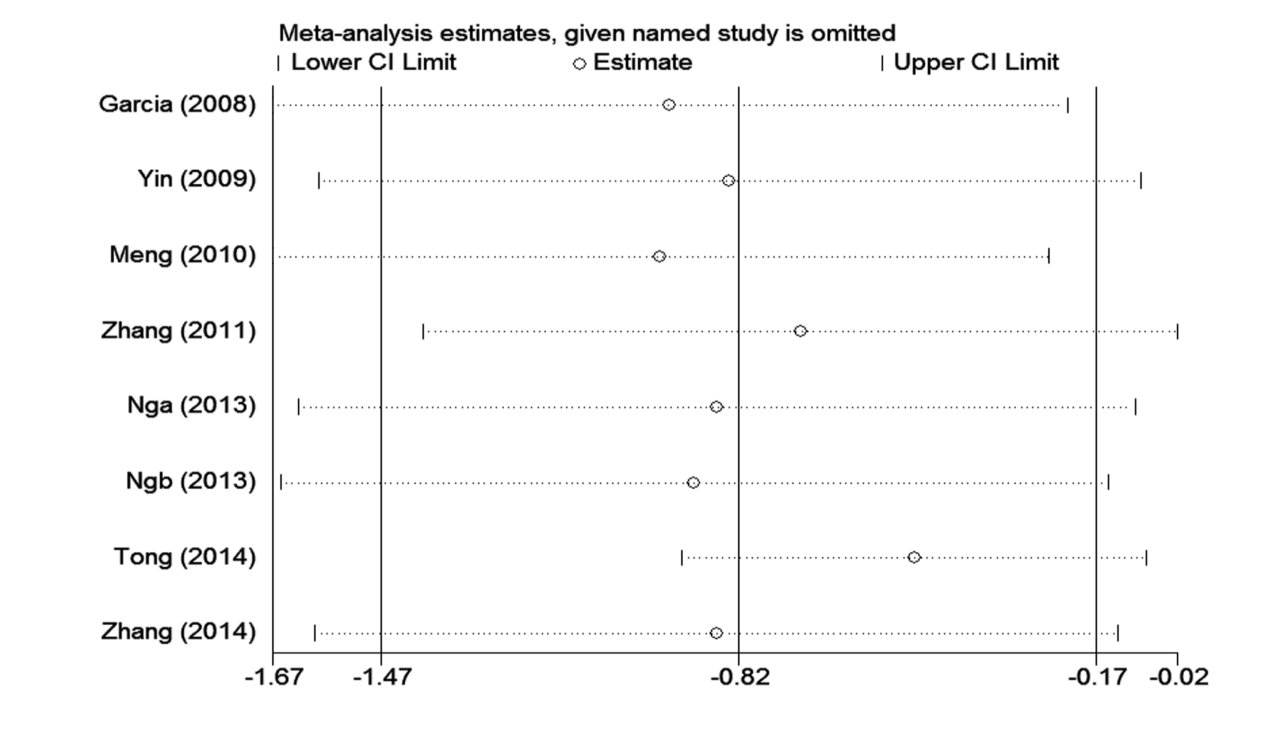


Supplemental file 2A. Sensitivity analysis for time to first flatus between acupuncture and control group.


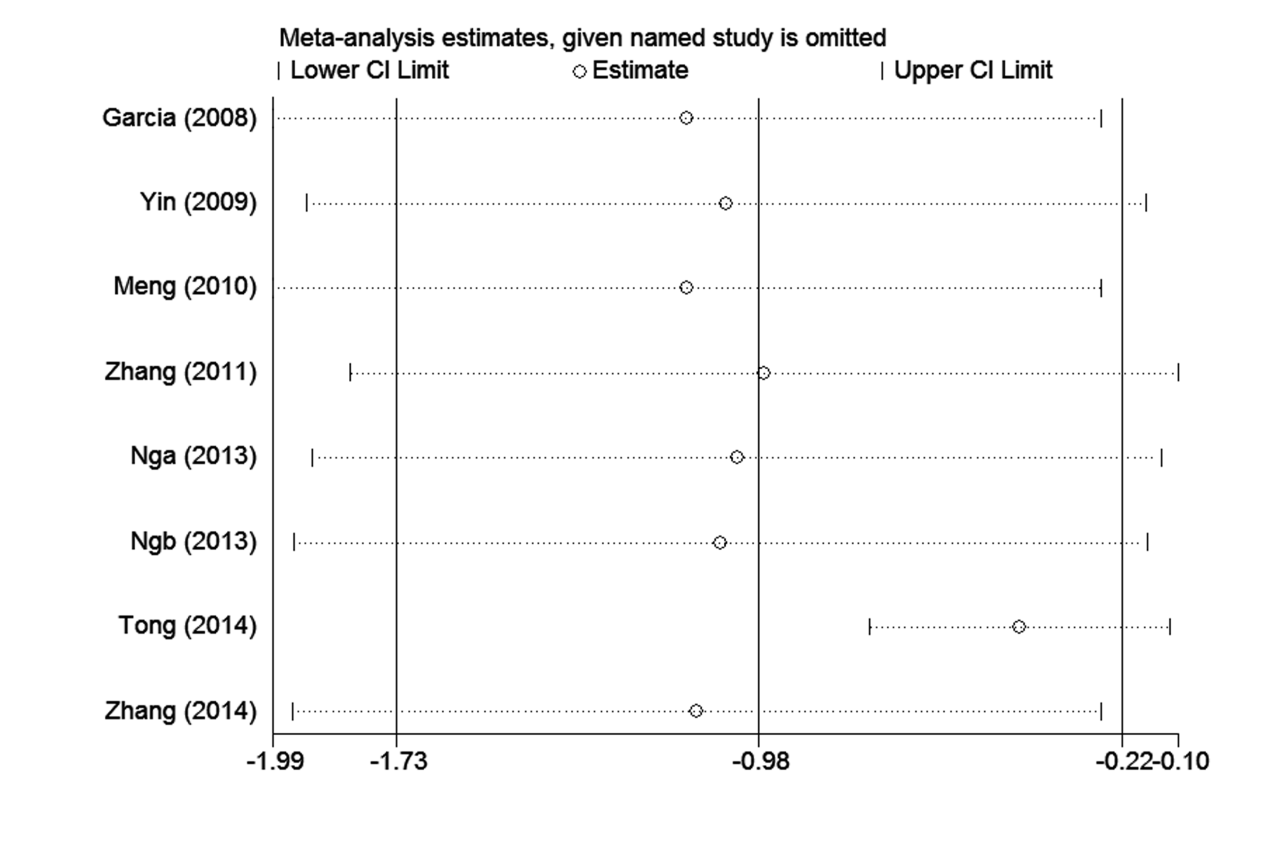


Supplemental file 2B. Sensitivity analysis for time to first defecation between acupuncture and control group.

**Supplemental file 2C-D.** Cumulative meta-analysis of the comparison between acupuncture and control group. **c** Time to first flatus; **d** Time to first defecation.


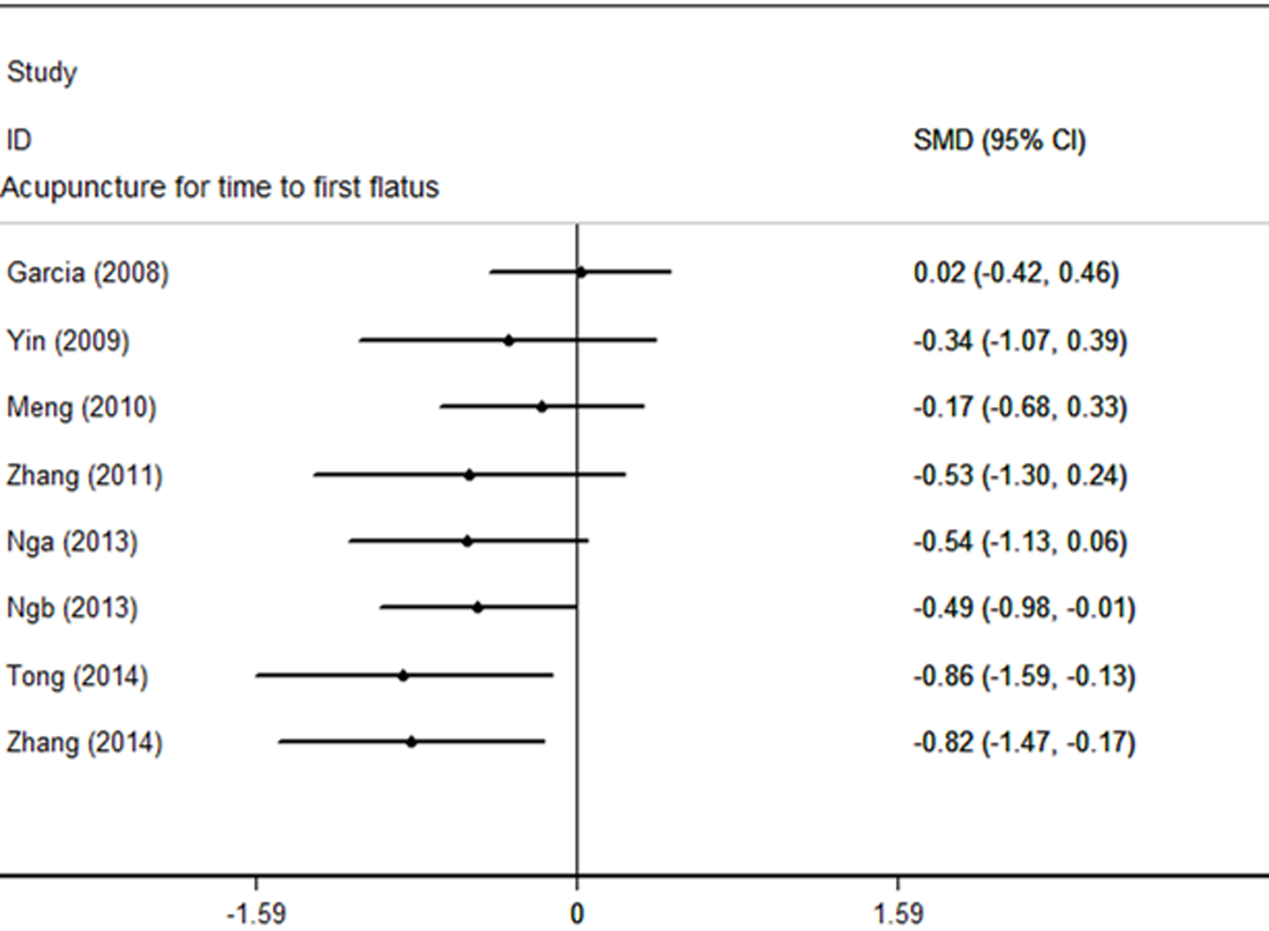


Supplemental file 2C: Cumulative meta-analysis of the comparison between acupuncture and control group: time to first flatus.


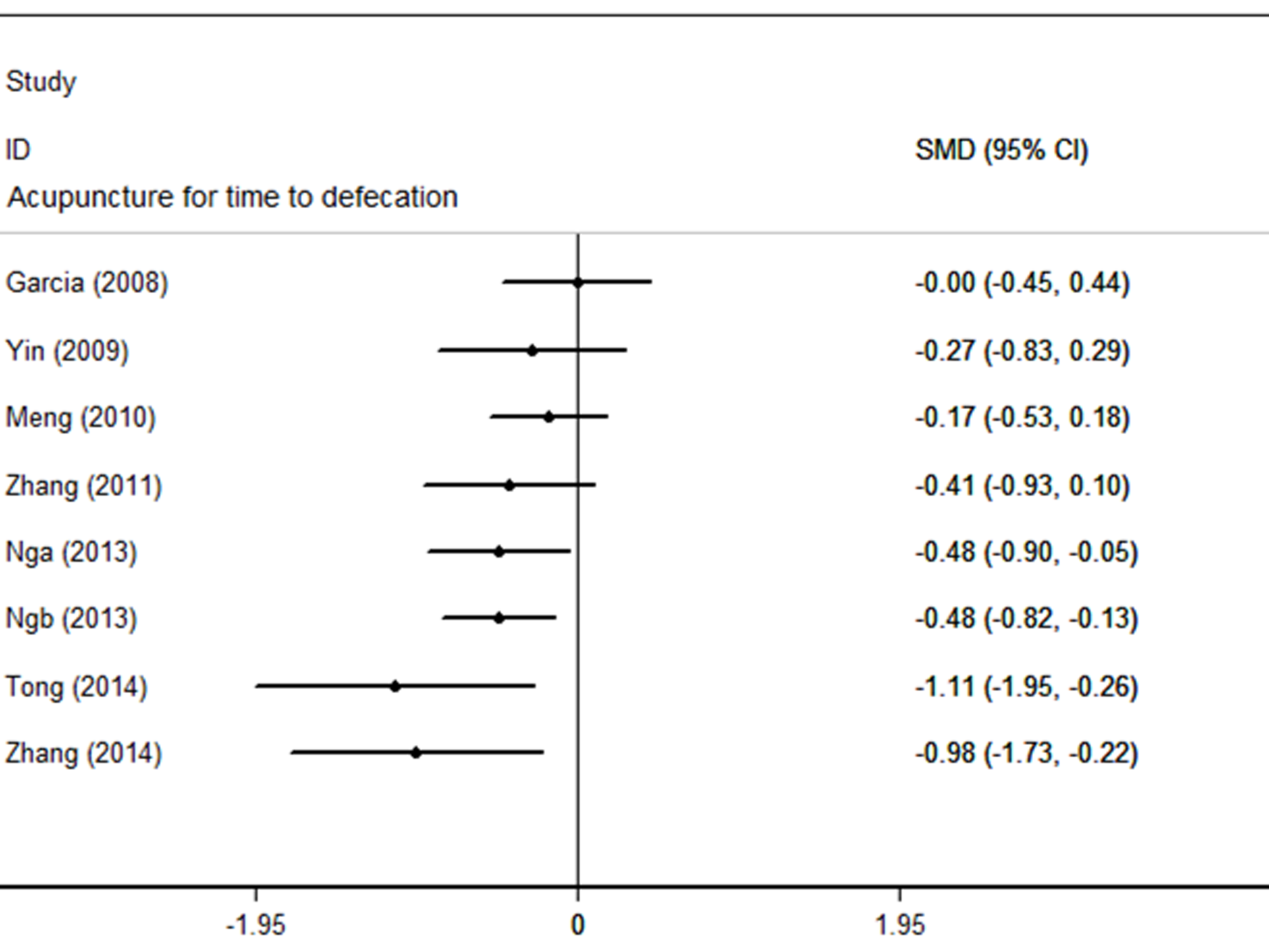


Supplemental file 2D: Cumulative meta-analysis of the comparison between acupuncture and control group: time to first defecation.

**Supplementary file3: Reporting quality of RCTs based on STRICTA.**

| Item | Detail | Garcia2008 | Yin  2009 | Meng2010 | Chao2010 | Zhang  2011 | Ng  2013 | Deng  2013 | Zhang  2014 | Tong  2014 | Hsiung  2015 | Comment |
| --- | --- | --- | --- | --- | --- | --- | --- | --- | --- | --- | --- | --- |
| 1.  Acupuncture rationale | 1a) Style of acupuncture (e.g. Traditional Chinese Medicine，Japanese，Korean，Western medical，etc) | Y | Y | Y | Y | Y | Y | Y | Y | Y | Y | Style of acupuncture or acupressure |
|  | 1b) Reasoning for treatment provided， literature sources，and/or consensus methods，with references where appropriate | Y | N | Y | Y | N | Y | N | N | N | N |  |
|  | 1c) Extent to which treatment was varied | N | Y | N | N | Y | Y | N | Y | N | N | “Y” is needed to state the detailed description |
| 2.  Details of needling | 2a) Number of needle insertions per subject per session (mean and range where relevant) | Y | Y | Y | Y | Y | Y | Y | Y | Y | Y | Number of treated  acupuncture and acupressure treatment per point per session |
|  | 2b) Names (or location if no standard name) of points used (uni/bilateral) | Y | Y | Y | Y | Y | Y | Y | Y | Y | Y |  |
|  | 2c) Depth of insertion，based on a specified unit of measurement | Y | N | N | Y | N | Y | N | Y | Y | N |  |
|  | 2d) Response sought (e.g. de qi or muscle twitch response) | Y | N | Y | Y | N | Y | Y | N | Y | Y |  |
|  | 2e) Needle stimulation (e.g. manual，electrical) | Y | Y | Y | Y | Y | Y | Y | Y | Y | Y | Procedure and technique for acupuncture and acupressure |
|  | 2f) Needle retention time | Y | Y | Y | Y | Y | Y | Y | Y | Y | Y | Time of treatment per point |
|  | 2g) Needle type (diameter，length，and manufacturer) | Y | Y | N | N | Y | Y | N | Y | N | N | Detailed feature |
| 3.  Treatment regimen | 3a) Number of treatment sessions | Y | Y | Y | Y | Y | Y | Y | Y | Y | Y |  |
|  | 3b) Frequency and duration of treatment sessions | Y | Y | Y | Y | Y | Y | Y | Y | Y | Y |  |
| 4.  Other components of treatment | 4a) Details of other interventions administered to the acupuncture group (e.g. moxibustion，cupping，herbs，exercises) | N | Y | N | Y | N | Y | Y | Y | N | Y |  |
|  | 4b) Setting and context of treatment，including instructions to practitioners，and information and explanations to patients | N | N | Y | N | N | N | N | N | N | N |  |
| 5.  Practitioner background | 5) Description of participating acupuncturists (qualification or professional affiliation，other relevant experience) | N | N | Y | Y | N | Y | N | Y | N | N |  |
| 6.  Control or comparator interventions | 6a) Rationale for the control or comparator in the context of the research question，with sources that justify this choice | N | N | N | Y | N | Y | Y | Y | N | N |  |
|  | 6b) Precise description of the control or comparator. If sham acupuncture or any other type of acupuncture-like control is used，provide details as for Items 1 to 3 above. | N | N | N | Y | N | Y | N | Y | N | N |  |

*N* not adequately reported; *Y* adequately reported ; *RCTs* randomized controlled trials; *STRICTA* standards for reporting interventions in clinical trials of acupuncture
